# High throughput screening identifies disulfide isomerase DsbC as a very efficient partner for recombinant expression of small disulfide-rich proteins in *E. coli*

**DOI:** 10.1186/1475-2859-12-37

**Published:** 2013-04-22

**Authors:** Hervé Nozach, Carole Fruchart-Gaillard, François Fenaille, Fabrice Beau, Oscar Henrique Pereira Ramos, Badreddine Douzi, Natalie J Saez, Mireille Moutiez, Denis Servent, Muriel Gondry, Robert Thaï, Philippe Cuniasse, Renaud Vincentelli, Vincent Dive

**Affiliations:** 1 CEA, iBiTec-S, Service d’Ingénierie Moléculaire des Protéines (SIMOPRO), CEA Saclay, Gif sur Yvette F-91191, France; 2 Architecture et Fonction des Macromolécules Biologiques (A.F.M.B.), UMR7257 CNRS, Université Aix-Marseille, Case 932, 163 Avenue de Luminy, Marseille cedex 9 13288, France; 3 CEA, iBiTec-S, Service de Pharmacologie et d'Immunoanalyse (SPI), CEA Saclay, Gif sur Yvette F-91191, France

**Keywords:** High throughput screening, Recombinant expression, Disulfide bonds, Cytoplasm, Disulfide-rich, Proteins, Fusion protein, *Escherichia coli*, Spectrometry, Mass, Matrix-assisted laser desorption-ionization

## Abstract

**Background:**

Disulfide-rich proteins or DRPs are versatile bioactive compounds that encompass a wide variety of pharmacological, therapeutic, and/or biotechnological applications. Still, the production of DRPs in sufficient quantities is a major bottleneck for their complete structural or functional characterization. Recombinant expression of such small proteins containing multiple disulfide bonds in the bacteria *E. coli* is considered difficult and general methods and protocols, particularly on a high throughput scale, are limited.

**Results:**

Here we report a high throughput screening approach that allowed the systematic investigation of the solubilizing and folding influence of twelve cytoplasmic partners on 28 DRPs in the strains BL21 (DE3) pLysS, Origami B (DE3) pLysS and SHuffle® T7 Express lysY (1008 conditions). The screening identified the conditions leading to the successful soluble expression of the 28 DRPs selected for the study. Amongst 336 conditions tested per bacterial strain, soluble expression was detected in 196 conditions using the strain BL21 (DE3) pLysS, whereas only 44 and 50 conditions for soluble expression were identified for the strains Origami B (DE3) pLysS and SHuffle® T7 Express lysY respectively. To assess the redox states of the DRPs, the solubility screen was coupled with mass spectrometry (MS) to determine the exact masses of the produced DRPs or fusion proteins. To validate the results obtained at analytical scale, several examples of proteins expressed and purified to a larger scale are presented along with their MS and functional characterization.

**Conclusions:**

Our results show that the production of soluble and functional DRPs with cytoplasmic partners is possible in *E. coli*. In spite of its reducing cytoplasm, BL21 (DE3) pLysS is more efficient than the Origami B (DE3) pLysS and SHuffle® T7 Express lysY *trxB*^*-*^*/gor*^*-*^ strains for the production of DRPs in fusion with solubilizing partners. However, our data suggest that oxidation of the proteins occurs *ex vivo*. Our protocols allow the production of a large diversity of DRPs using DsbC as a fusion partner, leading to pure active DRPs at milligram scale in many cases. These results open up new possibilities for the study and development of DRPs with therapeutic or biotechnological interest whose production was previously a limitation.

## Introduction

Small proteins containing disulfide bonds are versatile bioactive compounds that possess important pharmacological, therapeutic, and/or biotechnological values. There is a growing interest in the use of small disulfide-rich proteins (DRPs) as therapeutics, as they have several advantages over traditional small molecule drugs due to their high affinity and selectivity [1]. Small reticulated proteins have many applications, such as ion channel blockers for severe chronic pain treatment [2], as scaffolds for the transfer of hotspot active sites of bigger or more complex proteins [3,4], as antimicrobial and host-defense peptides [5] or molecular imaging agents [6]. In most cases, DRPs are composed of 20 to 120 residues and include between one to five disulfide bonds that are often crucial for activity and stability of these proteins. Oxidation of cysteine residues and proper disulfide pairing are indeed necessary for the correct spatial distribution of the key functional residues. Unfortunately, study and development of DRPs of therapeutic or biotechnological interest are still hampered by the difficulty to produce native and active proteins in sufficient amounts.

*E. coli* has many well-known advantages as a host for heterologous protein expression [7]. Various and complementary approaches have been described to produce native and soluble proteins in this bacterial host. In the past decade, several high throughput platforms have been used to identify optimal conditions for the soluble expression of proteins, notably by varying parameters such as fusion partners, strains or temperature [8-12]. Surprisingly, whereas several examples of successful expression of reticulated proteins in *E. coli* have been described [13-16], there is, to our knowledge, no study reporting parallel expression screening of many proteins containing various numbers of disulfide bonds.

Even if the production of various disulfide-bonded proteins in *E. coli* has previously been reported, expression of proteins with multiple disulfide bonds is still considered difficult [17]. As shown *in vitro* for the well-studied Bovine Pancreatic Trypsin Inhibitor (BPTI), the folding of disulfide-bonded proteins is often acquired through the accumulation of disulfide intermediates [18,19]. For some disulfide-rich proteins, oxidative folding generates heterogeneous populations of intermediates containing native but also non-native disulfide-bonded species, which require isomerization to reach the natively-folded oxidized state [20,21]. Thus, proteins with disulfide bonds are especially prone to aggregation due to possible mispairing of cysteine residues or undesirable intermolecular disulfide bonds. When overexpressed in bacteria with strong promoters, recombinant proteins often tend to misfold and accumulate as insoluble aggregates or inclusion bodies [22]. In many cases, the difficulty in reaching native conformation increases with the number of cysteine residues due to the number of possible isoforms, but also with the complexity of disulfide bond patterns. Failure to reach a native and stable conformation results, in most cases, in either protein aggregation or proteolytic degradation [23].

In past years, many approaches have been developed to promote the formation of disulfide bonds and the native folding of disulfide-rich proteins [17]. Exporting the proteins to the *E. coli* oxidizing periplasm is an intuitive strategy [24,25], as folding of proteins can be assisted by the disulfide bond formation system [26-28]. However, secretion of proteins to the periplasm often leads to low protein levels [28], probably because of the limited periplasmic volume combined with an insufficient capacity of the translocation machinery [29]. Because of these limitations, many strategies consider expression in the *E. coli* cytoplasm, even for proteins containing disulfide bonds. Oxidation of cysteine thiols in the reducing cytoplasm of wild-type *E. coli* is described as unfavorable for both thermodynamic and kinetic reasons [17,23]. To overcome this issue, engineered strains like Origami (DE3) pLysS with an oxidative cytoplasm were developed [30,31]. These strains contain deletions of both glutathione and thioredoxin reductase genes (*gor*^*-*^*, trxB*^*-*^) along with an additional mutation in the peroxiredoxin gene *ahpC* necessary to restore growth. Some studies indicate that these strains enhance the accumulation of oxidized proteins in the cytoplasm [17,32,33]. Several other engineered strains with altered reducing pathways are described to improve production levels of disulfide-bonded proteins [34,35]. The amount of oxidized protein can be further enhanced by co-expression of redox-active enzymes like thioredoxin (Trx), Trx mutants or DsbC in the cytoplasm of *trxB*^*-*^/*gor*^*-*^strains [30,36]. Thioredoxin mutants are of particular interest because the two residues included in the dicysteine active site (CxxC) are effecting their oxidoreductase activities. Indeed, mutating these two amino acids shifts the activity from a reductase to an oxidase in oxidative environments. These characteristics are described as very useful for the production of proteins with disulfide bonds [30,37].

In addition to those approaches for promoting the formation of disulfide bonds, the question of the solubilization of the protein of interest and its folding intermediates has to be addressed. Many fusion tags are described to enhance protein solubility *in vivo*[38-40]. Several proteins have been extensively used as fusion partners like glutathione S-transferase (GST) [41], maltose binding protein (MBP) [42], double Z-domain from staphylococcal protein A (ZZ) [43] or Gb1-domain from protein G (Gb1) [44]. Some other fusion partners not only have an important solubilizing effect but also redox properties, which could be beneficial for disulfide bond formation. From this perspective, thioredoxin is not only an oxidoreductase but also one of the most potent solubilizing partners available [45]. For the above purpose, the proteins DsbA and DsbC, although not as yet explored, could also be of interest as solubilizing partners for cytoplasmic expression, if expressed without their signal peptides. Indeed, disulfide isomerase DsbC retains a foldase and chaperonin activity when used in co-expression in the cytoplasm [36,46]. Nevertheless, even if several publications report the positive effect of fusion partners or use of specific strains on the recombinant expression of specific DRPs, no general study has yet explored the efficacy of these systems on a large diversity of DRPs in order to document general rules for this protein family.

Here we report the results of a high throughput screen for the soluble expression of small proteins with disulfide bonds in the *E. coli* cytoplasm. Given a set of DRPs; 28 different proteins of variable size (from 25 to 122 aa) with two to five disulfide bridges, the objective of this study was to identify the best fusion partners and strains to provide access to milligram amounts of oxidized and functional recombinant DRPs.

## Results

### Study set up

**Figure 1:**
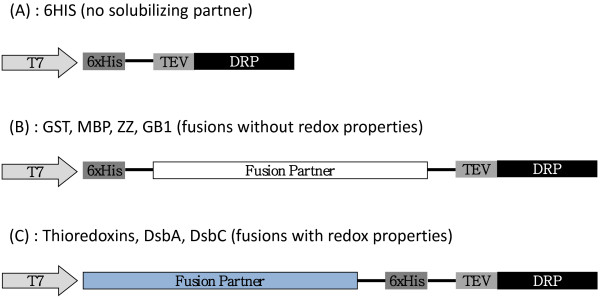
**Design of the expression plasmids.** All plasmids carry a T7 promoter/terminator, a 6HIS tag for nickel affinity purification, a Tobacco Etch Virus (TEV) cleavage site and a gene encoding a disulfide-rich protein (DRP). The 6HIS tag is N-terminal for the 6HIS plasmid **(A)** and for fusions without redox properties **(B)**. The 6HIS tag is placed between the fusion partner and the TEV site for fusion with redox properties **(C)**. Non-redox fusion partners are represented in white boxes while redox fusions are represented in blue. The same representation of the redox properties of the fusions appears throughout the figures.

**Figure 2:**
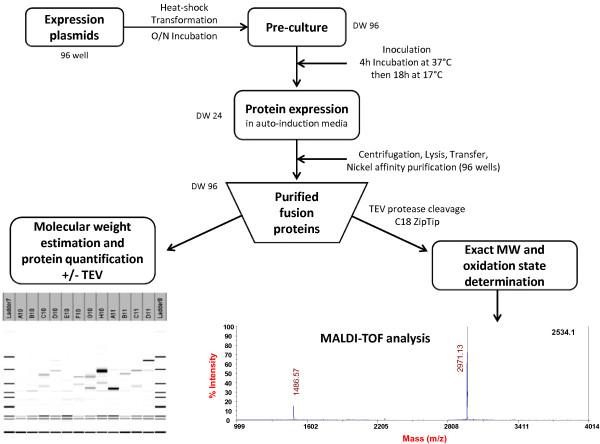
**Schematic representation of the high throughput expression screening procedure.** For details, see Materials and methods.

We have selected 28 targets (Additional file 1: Table S1 Data), representing six different folds (ICK, 3FT, Kunitz, Kazal type, α/β, 3_10_ helix). This set includes proteins for which functional tests are available to allow the assessment of the correct folding of the targets based on the preservation of their native binding properties. All these proteins have an even number of cysteine residues that are all involved in a disulfide bond. Twelve different fusion partners targeting the protein into the cytoplasm were selected in our study (Additional file 2: Table S2 Data). In every case a hexa-histidine (6HIS) tag was introduced to enable the downstream purification of the fusion proteins using immobilized-nickel affinity chromatography. A TEV (Tobacco Etch Virus) protease cleavage site (ENLYFQ/G) was introduced to enable removal of the fusion partner. In addition to the 6HIS affinity tag alone which serves as a reference, the 11 other fusion partners selected in our study include glutathione S-transferase (GST) [41], maltose binding protein (MBP) [42], double Z-domain from staphylococcal protein A (ZZ) [43], Gb1-domain from protein G (Gb1) [44], thioredoxin (Trx) and four of its active site mutants [30], the disulfide oxidoreductase DsbA and the disulfide isomerase DsbC from *E. coli*[47] without their signal peptides to allow cytoplasmic expression. The four mutants of Thioredoxin are Trx-A with a DsbA-like active site (CPHC), Trx-C with a DsbC-like active site (CGYC), Trx-G with a glutaredoxin-like active site (CPYC) and Trx-P with a PDI-like active site (CGHC). All expression vectors were constructed using Gateway cloning technology (Invitrogen) [48]. A schematic representation of each vector can be found in Figure 1. To benchmark the effect of the strains on the yield of DRPs, three different *E. coli* expression strains were used in this study. BL21 (DE3) pLysS with a reducing cytoplasm, the Origami B (DE3) pLysS with a more oxidizing cytoplasm and finally the SHuffle® T7 Express lysY which are similar to the Origami B (DE3) pLysS (oxidizing cytoplasm) but was engineered to express also cytoplasmic DsbC [36]. The screening scheme (Figure 2) is a slightly modified version of the high throughput protocols described in detail elsewhere ([9,49]). The culture and expression were performed in auto-induction media [50]. After nickel affinity purification, systematic analysis of the Labchip GXII electropherograms was performed to determine the concentration of the purified proteins and compare the apparent molecular weight of the purified fusion proteins with their expected theoretical molecular weight. After incubation with the protease TEV to remove the fusion partner, the cleavage was confirmed by analysis on the Labchip GXII and the accurate mass and oxidation state of each peptide were determined by mass spectrometry. Whenever possible, functional assays were performed to demonstrate the accuracy of their folding.

### Influence of the fusion partner and strains on soluble expression

**Figure 3:**
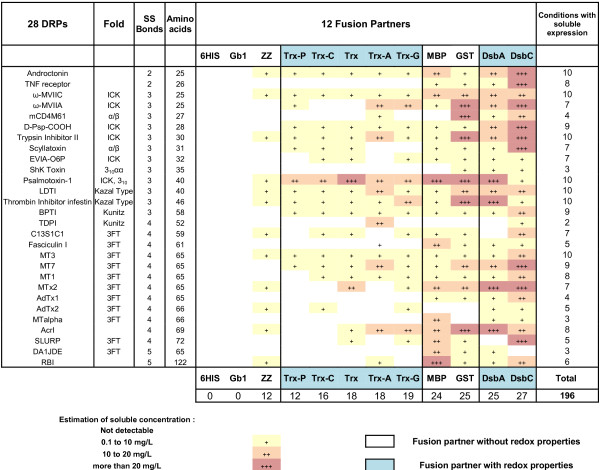
**Impact of 12 different fusion partners on the solubility of DRP in the strain BL21 (DE3) pLysS.** The DRPs are ordered by increasing number of disulfide bonds and amino acids. The protein fold (where known) is indicated. The fusions are ranked according to the number of soluble DRPs detected. When the number of soluble conditions is the same they are ranked according to the number of DRP fusions produced above 10 mg/L. These rankings are kept throughout Figure 3 to 5.

**Figure 4:**
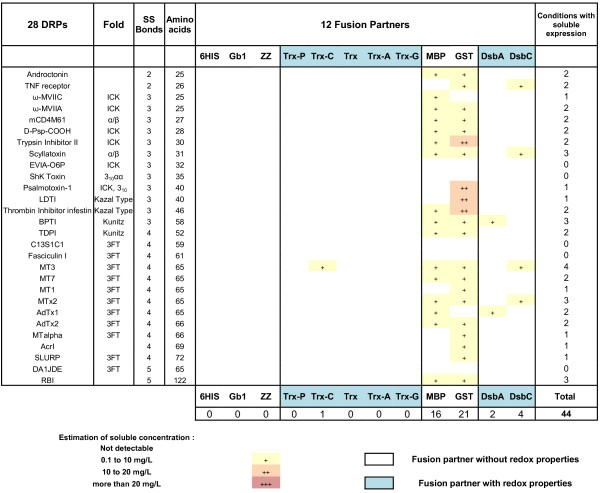
Impact of 12 different fusion partners on the solubility of DRP in the strain Origami B (DE3) pLysS.

**Figure 5:**
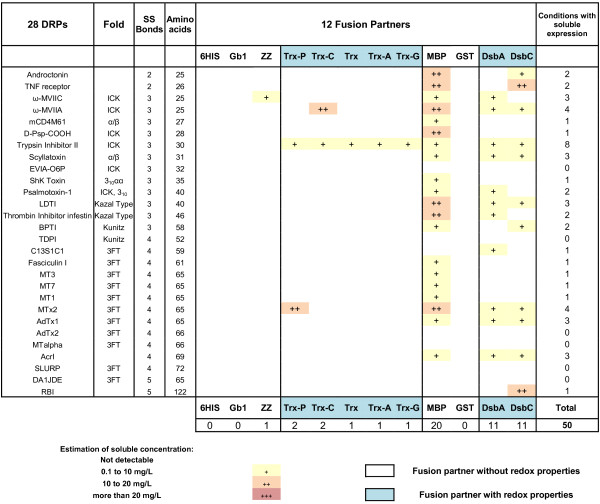
Impact of 12 different fusion partners on the solubility of DRP in the strain SHuffle® T7 Express lysY.

An exhaustive analysis of the 1008 conditions tested (28 proteins x 12 fusion partners x 3 strains) was performed in parallel (Figures 3, 4 and 5). The amounts of soluble fusion proteins were estimated after the nickel purification using the Caliper GX II semi quantitative method (See Materials and Methods). The experiments on the 1008 cultures were performed in duplicates. The soluble level was in good agreement between the two experiments and therefore the soluble yields were averaged between the two experiments and ranked according to four categories of soluble level: not detected, soluble from 0.1 to 10 mg/L, 10 to 20 mg/L and greater than 20 mg/L. The limit of 20 mg/L was dictated by the volume of nickel beads used in the expression screening protocol (50 μL); a volume suitable to detect low expressing proteins (> 0.1 mg/L of culture with Caliper GX II detection) but saturated with soluble levels above 20 mg/L [9,49]. While the expression screening protocol used in this study gave a good indication of the soluble level and while it allowed the ranking of the impact of the fusion based on soluble yields, it was not designed to discriminate between several conditions that would all give soluble levels above 20 mg/L. In this study, at preparative scale, the purified fusion proteins were sometimes above 200 mg/L (see “Functional characterization”). This prompted us to design a modified expression screening protocol (based on 200 μL of beads [49]) that was validated on the high expressing fusions of this study and confirmed the ranking obtained with 50 μl of beads (data not shown).

**Figure 6:**
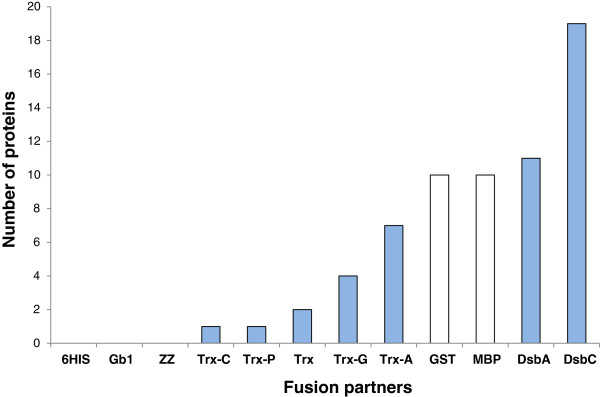
**Impact of the fusion partner on the number of soluble proteins.** White: Non-redox fusions. Blue: redox fusions. Bars represent the number of soluble DRPs produced above 10 mg/L of culture in fusion with the specified partner. The strain used is BL21 (DE3) pLysS.

Out of 336 constructs in BL21 (DE3) pLysS, soluble expression of fusion proteins was observed in the milligram range of protein per liter of culture in 196 cases (58%), with correct apparent molecular weights (Figure 3). Fusion with MBP, GST, DsbA and DsbC partners resulted in soluble expression of most of the DRPs (more than 85% for MBP, GST and DsbA and more than 95% for DsbC). Fusion with the different thioredoxins or ZZ was successful, but less effective, with soluble expression rates ranging from 40% to 70%. Soluble expression was observed neither with the sole 6HIS tag nor with the fusion Gb1, where DRPs in fusion were often detected as inclusion bodies (data not shown). As shown in Figures 3 and 6, the four best solubilizing fusions (MBP, GST, DsbA and DsbC) not only gave access to a wider variety of soluble DRPs but also gave higher amounts of soluble protein for the 12 fusions tested. Many DRPs were expressed with significant expression levels when fused with those four partners. Specifically, 18 DRPs out of 28 were expressed with expression levels greater than 10 mg/L of fusion protein using DsbC as a fusion partner (Figure 6). This number is significantly higher with DsbC than for any of the other fusion partners tested (Figure 6).

In contrast, soluble expression was observed in only 44 conditions for the Origami B (DE3) pLysS strain (Figure 4) and in only 50 conditions in the strain SHuffle® T7 Express lysY (Figure 5) as compared to 196 with BL21 (DE3) pLysS strain (Figure 3). The estimated amounts of soluble fusion proteins obtained in both strains were almost always lower or in the best cases only similar to those produced in the same condition in BL21 (DE3) pLysS. For the Origami B (DE3) pLysS, the fusion with MBP or GST resulted in soluble expression for the majority of DRPs (57% and 75% respectively) while soluble expression with thioredoxins, DsbA and DsbC were only observed in rare cases (< 1%, <10% and <15% respectively). Out of the 44 soluble conditions, the soluble level was mainly achieved at very low concentrations (90% of the cases are below 10 mg/L) except in the cases of four GST fusions.

For the SHuffle® T7 Express lysY, the fusion with MBP was the only one allowing the soluble expression for a majority of DRPs (71%). The successful soluble expression with ZZ and thioredoxins (below or around 5%) was very rare while the fusion with DsbA and DsbC (40%) was far from the same as cultures in BL21 (DE3) pLysS, but significantly better than in Origami B (DE3) pLysS. Out of the 50 soluble conditions, the soluble level was mainly achieved at very low concentrations (78% of the cases are below 10 mg/L). Most of the 22% that were expressed between 10 to 20 mg/L were expressed as MBP fusions.

In our set-up, the differences observed between BL21 (DE3) pLysS versus Origami B (DE3) pLysS and SHuffle® T7 Express lysY were important, both in terms of number of soluble constructs and yields. In particular, of the 28 DRPs the soluble yield obtained with the Origami B (DE3) pLysS or the SHuffle® T7 Express lysY was never better than the yield of the equivalent condition in BL21 (DE3) pLysS. For these reasons, we have chosen to focus on the production of DRPs using only the strain BL21 (DE3) pLysS in the rest of the study.

### Influence of the fusion partner on oxidation

**Figure 7:**
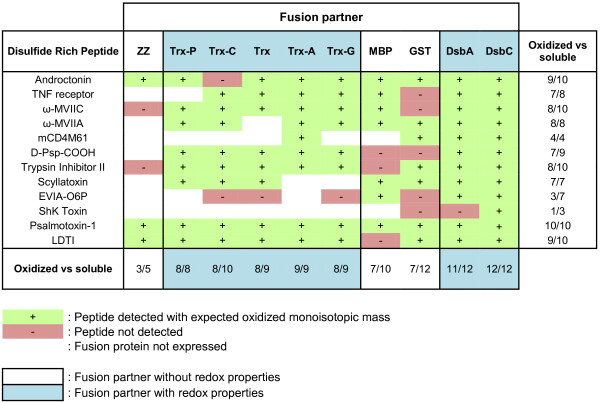
MALDI-TOF detection of oxidized DRPs using different fusion partners.

**Figure 8:**
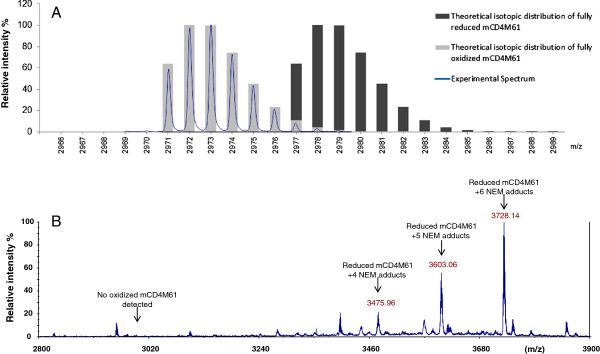
**MALDI-TOF analysis of mCD4M61. **Panel **A:** Comparison of the experimental MALDI-TOF spectrum obtained for the mCD4M61 with the theoretical isotopic distributions of fully reduced and fully oxidized mCD4M61. The mCD4M61 DRP was produced using DsbC as fusion partner. Panel **B:** Experimental MALDI-TOF spectrum obtained for the mCD4M61 using N-Ethylmaleimide as an alkylating agent during cell lysis.

Next, an extensive analysis of the oxidation state of the DRPs was performed by MALDI-TOF. As MALDI-TOF analyses with isotopic resolution are limited to very small proteins, only the 12 DRPs with a molecular mass lower than 6500 Da could be analyzed (Figure 7). All the constructs yielding soluble fusion proteins for those 12 DRPs in the strain BL21 (DE3) pLysS (96 out of 120) were submitted to TEV cleavage, desalting and MALDI-TOF analysis. Amongst these 96 constructs, the monoisotopic masses corresponding to the correctly oxidized species were detected in 81 cases (See Figure 7: conditions in green). In all analyses performed, the MALDI-TOF spectra matched the theoretical isotopic distribution for the DRP with fully oxidized cystines (see Figure 8A: experimental MALDI-TOF spectrum obtained for mCD4M61). The reduced forms of the proteins could not be detected (data not shown), probably due to aggregation or precipitation. Oxidized proteins were detected in a large majority of cases when fused to partners with redox activity (between 70 to 100% for thioredoxin and its mutants, 92% for DsbA and 100% for DsbC, see Figure 7). This percentage is lower when fusions have only a solubilizing role (60% for ZZ, 70% for MBP, 58% for GST). To access the oxidation state of DRPs larger than 6500 Da, we also analyzed selected DRPs in fusion with their solubilizing partner with an LTQ-Orbitrap mass spectrometer. As DsbC was by far the best fusion partner among those investigated, the exact masses of 25 DsbC-DRP fusions were determined. These analyses demonstrated that out of these 25 samples, 17 fusion proteins had molecular masses in agreement with the expected masses considering fully oxidized cystine residues, with a mass difference between the observed and expected masses of less than 0.7 Da on average (Additional file 3: Table S3).

As mass spectrometry analyses revealed that oxidized DRPs were detected in many cases, we tried to determine if the formation of disulfide bonds occurred in the cytoplasm of BL21 (DE3) pLysS or during the subsequent extraction and purification steps. To elucidate this question, we investigated the particular case of mCD4M61 produced in fusion with DsbC. A thiol-blocking agent, N-ethyl maleimide (NEM) was introduced in the lysis buffer to prevent *ex vivo* oxidation. NEM readily reacts with free cysteine residues, thus yielding a 125.13 Da mass increment per alkylated cysteine. However, as NEM does not react with cystines involved in disulfide bridges, NEM treatment would leave *in vivo* oxidized mCD4M61 unmodified. After lysis and incubation in the presence of 100 mM NEM, purification and TEV cleavage were performed as in the previous experiments. MALDI-TOF analysis revealed the presence of a major peak at *m/z* 3728.14 in perfect agreement with the calculated value for the fully reduced DRP bearing 6 NEM adducts (*m/z* 3728.13), i.e. 3 fully reduced and alkylated disulfide bridges (Figure 8B). Analysis in the presence of NEM also revealed two other accompanying peaks at *m/z* 3603.06 and 3475.96 corresponding to reduced and partially alkylated proteins bearing 5 and 4 NEM adducts, respectively. The fully oxidized peptide could not be detected in the presence of NEM suggesting that the formation of disulfide bonds in the mCD4M61 mostly occurred *ex vivo* (Figure 8B).

### Functional characterization

Functional characterization through an activity test is one of the best methods to discriminate DRPs with their native fold and correct disulfide patterns from misfolded isoforms. Purifications from larger scale cultures using BL21 (DE3) pLysS were performed on six selected DRPs (mCD4M61, Trypsin Inhibitor II, LDTI, Thrombin Inhibitor Infestin, BPTI and MT7) for which functional tests are available. DsbC was chosen as a fusion partner because it gave the highest yields of soluble and oxidized protein in the analytical screen. Very large amounts of DsbC-DRP fusion proteins were obtained after nickel affinity purification, ranging from 90 mg for the mCD4M61 fusion to 291 mg for the Trypsin Inhibitor II fusion. Fusion proteins were digested with TEV protease to release the DRPs. After purification to homogeneity using reverse phase HPLC (i.e. >95%), all DRPs were produced in quantities varying between 0.8 and 12 mg/L (Table 1), except for the muscarinic toxin MT7 for which only a few micrograms were obtained. The functional tests of these 6 proteins revealed that they were fully active (Table 1) with inhibition constants similar to the values reported in the literature [51-55], leading us to conclude that these DRPs were produced with their natively folded structures.

**Table 1:** Functional validation of purified DRPs

								**Functional test**	
**Disulfide Rich Peptide**	**Fold**	**SS**	**DsbC fusion produced (mg/L)**	**Theoretical DRP (mg/L)**	**Purified DRP (mg/L)**	**DRP yield**	**Detected mass**	**Test**	**Observed vs Expected**
mCD4M61	α/β	3	90	8.9	**3.5**	39%	2972.0 ± 0.5	ELISA	69 nM (32 nM)
Trypsin Inhibitor II	ICK	3	291	30.1	**0.9**	3%	3124.4 ± 0.3	Trypsin Inhibition	1.0 nM (0.3 nM)
LDTI	Kazal Type	3	240	35.0	**12.0**	34%	4572.1 ± 0.1	Trypsin Inhibition	1.8 nM (1.8 nM)
Thrombin Inhibitor Infestin	Kazal Type	3	120	20.2	**0.8**	4%	5507.7 ± 0.2	Trypsin Inhibition	1.8 nM (2 nM)
BPTI	Kunitz	3	180	35.1	**3.0**	9%	6567.6 ± 0.1	Plasmin inhibition	0.4 nM (0.14 nM)
MT7	3FT	4	160	34.7	**0.007**	< 0.03%	7529.9 ± 0.6	binding mAChR	5 pM (29 pM)

Purification from 1 liter culture of DsbC-DRP fusion in BL21 (DE3) pLysS strain.

## Discussion

We have developed a high throughput expression screen that enabled us to assess the solubility of DRPs fused to a large number of partners. We used it to study the impact of twelve fusion tags and three expression strains on the soluble level of 28 DRPs. Here, we show that a general scheme for bacterial expression in the cytoplasm of *E. coli* of genes coding for DRPs could be successfully implemented. Our approach consists of (i) cloning by recombination of genes in our set of vectors, (ii) culturing of *E. coli* in auto-induction media for the expression of fusion proteins in the cytoplasm (iii) lysis of bacteria, nickel affinity purification and analysis of fusion proteins, (iv) digesting with TEV and MALDI-TOF detection of oxidized DRPs. In the present study, we identified the conditions leading to soluble expression of the 28 selected DRPs in *E.* coli. Amongst the 1008 conditions tested in duplicates (28 proteins x 12 fusions x 3 strains), 196 conditions allowed soluble expression in the strain BL21 (DE3) pLysS, whereas only 44 and 50 conditions gave soluble expression for the strain Origami B (DE3) pLysS and SHuffle® T7 Express lysY, respectively.

The four-fold difference between Origami B (DE3) pLysS or SHuffle® T7 Express lysY and the BL21 (DE3) pLysS strain cannot be explained by medium composition, expression plasmids or cultivation differences, as all tests were run in the same conditions. Furthermore, thanks to the removal of the antibiotics used to maintain the *trxB*^*-*^*/gor*^*-*^ genomic mutations in Origami (DE3) pLysS and SHuffle® T7 Express lysY, the growth rate of the three strains were very similar. An increased lag phase was observed with the Origami B (DE3) pLysS and SHuffle® T7 Express lysY but at harvesting time, in stationary phase, the optical density of the 672 cultures was in all cases around 12 (with less than 10% difference) and therefore the yield of soluble expression was considered as normalized by bacterial biomass. The low number of soluble constructs in Origami B (DE3) pLysS and SHuffle® T7 Express lysY is directly associated with low production levels. In practically all cases these two strains produced lower amounts of soluble fusion protein than BL21 (DE3) pLysS and out of the 28 DRPs, none would be favorably produced in Origami B (DE3) pLysS or SHuffle® T7 Express lysY as opposed to BL21 (DE3) pLysS. The low production level of DRPs in in Origami B (DE3) pLysS or SHuffle® T7 Express lysY was confirmed with scale-up tests (unpublished observations).

Strikingly, soluble expression of DRP fusions in Origami B (DE3) pLysS or SHuffle® T7 Express lysY are mostly observed with fusion partners lacking redox activity like GST or MBP for Origami B (DE3) pLysS and MBP for the SHuffle® T7 Express lysY. Only a few constructs were soluble using redox-active fusion partners (Trx, Trx mutants, DsbA and DsbC) and at levels below the equivalent BL21 (DE3) pLysS cultures. In spite of this, SHuffle® T7 Express lysY performed better with DsbA and DsbC than Origami B (DE3) pLysS. These results contrast with the good results of redox-active fusion partners in the strain BL21 (DE3) pLysS. This is rather unexpected since redox-active proteins such as thioredoxin or DsbC are often described as enhancing disulfide bond formation in the cytoplasm of Origami B (DE3) pLysS or equivalent strains either when co-expressed [36,56] or used as a fusion partner [57]. The low production levels in Origami B (DE3) pLysS and SHuffle® T7 Express lysY strains might be linked to the fact that these strains are not as robust as other strains like BL21 (DE3) pLysS, often having altered growth parameters compared to other *E. coli* strains [9,58]. It is important to note that the *trxB*^*-*^*/gor*^*-*^ strains only have the reducing pathways of the cytoplasm disrupted. This means that once a disulfide bond is formed, it is more likely to be retained than in a reducing cytoplasm, however, it does not necessarily make disulfide bond formation more efficient. Firstly, there are no added catalysts for *de novo* disulfide bond formation present to promote disulfide bond formation in the first place. In addition, while oxidation in the non-reducing cytoplasm can occur by the transfer of disulfide bonds to folding proteins from oxidized thioredoxin (which can no longer be reduced by the canonical pathway due to the *trxB*^*-*^*/gor*^*-*^ mutation), this is relatively inefficient and slow [59]. It has been suggested that correct folding in a *trxB*^*-*^*/gor*^*-*^ background is to the detriment of yield [60] and accordingly, these strains have been reported to often produce very low yields [61,62]. In fact, even with the co-expression of DsbC to catalyze disulfide isomerization, yields of oxidized correctly folded protein can only be improved if (i) the expressed protein is soluble (perhaps necessitating the use of additional solubilizing fusion tags), and (ii) if the disulfide bonds are already formed (either spontaneously or by interaction with other disulfide bond donor proteins, including DsbC itself). These reasons may explain why soluble yields from SHuffle strains have not been improved beyond those found in Origami strains for the expression of certain proteins [58]. It must be noted however, that use of Origami B (DE3) pLysS or SHuffle® T7 Express lysY might necessitate screening of additional parameters such as medium, induction conditions, temperatures [36] or protein co-expression that were not investigated in our study. However, in our set-up, the difference observed between BL21 (DE3) pLysS and Origami B (DE3) pLysS or SHuffle® T7 Express lysY was significant and we have chosen to focus only on the strain BL21 (DE3) pLysS.

In contrast to Origami B (DE3) pLysS or SHuffle® T7 Express lysY, the success rates of DRPs expressed as fusions in the cytoplasm of BL21 (DE3) pLysS was high (196/336). For every DRP of interest, multiple soluble expression conditions were observed. Our procedure yields relatively high quantities of fusion proteins after nickel affinity purification: approximately 20% of the conditions tested led to estimated quantities greater than 10 milligrams per liter of culture (65/336). Quantities of fusion proteins are higher with MBP, GST, DsbA and DsbC than for the other fusion partners. Use of thioredoxin and thioredoxin mutants could be an interesting option, because of their lower molecular weight compared to the other fusion partners (15 kDa vs 25–40 kDa), thereby providing a greater proportional yield of DRPs to fusion tag [13]. Even so, from the data collected here, the impact of the thioredoxin mutations is difficult to appreciate, both in terms of solubilization and quantities of fusion.

To investigate the redox states of the DRPs when produced in fusion, we have coupled the solubility screen with MS detection of the proteins whenever possible. MALDI-TOF analyses revealed that detectable amounts of oxidized proteins can be cleaved off from the fusion partners in a very high number of cases using BL21 (DE3) pLysS as a production strain. These observations raised the question of whether the disulfide bond formation occurred *in vivo* or *ex vivo*. The *ex vivo* formation of disulfide bonds is a hypothesis also suggested previously by Tedford *et al.*[63] based on the equivalent oxidized production of a single spider toxin in BL21 versus a *trx*^*-*^ strain. Thus, to test this hypothesis, the disulfide bond status of mCD4M61 fused with DsbC was examined by subjecting the lysate to addition of N-ethylmaleimide at the moment of cell lysis. In contrast with the untreated samples, oxidized mCD4M61 could not be detected after purification and TEV cleavage, indicating that the disulfide bond formation probably occurred *ex vivo*. The strain BL21 (DE3) pLysS allows the production of high amounts of DRPs fused with different partners but its cytoplasm probably remains an unfavorable environment for the formation of disulfide bonds. In contrast, the purification steps after cell lysis probably provide a more favorable environment for the DRP to reach an oxidized state. Purification steps not only remove the reducing pathway components that could hamper the disulfide bond formation but are also performed at pH 8, rendering protein thiols (typically p*K*_a_ ∼ 8–9) very reactive. The buffer also has higher concentrations of molecular oxygen that can act as final electron acceptors and thus be the driving force leading to the oxidation of the peptide.

The data collected suggest that the redox properties of the fusion partner might have an effect on the folding of the DRPs. First, we have shown that either redox-inactive (MBP, GST) or redox-active (DsbC, DsbA) fusion partners are able to produce significant quantities of fusion proteins for many DRPs. However, MALDI-TOF experiments revealed that constructs for which oxidized peptide could not be detected are, in most cases, fusions without redox properties (e.g. MBP, GST). In contrast, most oxidized DRPs are detected when thioredoxins, DsbA or DsbC were used as fusion partners. These observations support the hypothesis that redox activity of the carrier has an influence on the folding of the DRP, even if oxidation occurs *ex vivo*. When using fusion partners with redox activity, the partner could not only improve the solubility of the folding intermediates but could also assist the DRPs to reach their native oxidized form. To further confirm this hypothesis, it could be interesting to investigate the effect of inactive redox variants of those partners on the yield of active DRP.

Under our criterions, use of DsbC as a fusion partner in the strain BL21 (DE3) pLysS was the most potent combination tested. Indeed, use of DsbC yielded soluble fusions for almost all the DRPs (27/28) but also has the strongest effect on the quantity of fusions produced. When DsbC is used as a fusion partner, more than 67% of the constructs (18/28) are produced with yields exceeding 10 mg/L at the screening scale. The true magnitude of those yields becomes even more apparent when proteins are expressed at larger scale (1 liter) with up to 290 mg/L for the DsbC fusions. Foremost, mass spectrometry experiments also revealed that DsbC generates oxidized folded DRPs in a significant number of cases. The favorable results obtained with DsbC can be explained by its excellent solubilization potential but most importantly by its isomerase and chaperonin activities [17], which are a considerable advantage to assist DRPs to reach their native active state. Most importantly, depending on the DRP properties, the proportion of folded DRP can be very significant as attested by the good yields obtained for scale-up production and purification using DsbC as fusion partner (e.g. LDTI, BPTI, mCD4M61, Table 1).

Only a few articles available in the literature report the successful production of oxidized DRPs in the *E. coli* cytoplasm without the use of *trxB*^*-*^*/gor*^*-*^ strains or *in vitro* refolding steps. Bogomolovas *et al.* reported the production of active viscotoxin A3 containing three disulfide bonds using thioredoxin as a fusion partner [13]. Similarly, Mac *et al.* reported production of Endothelin-1 containing two disulfides using thioredoxin [64], while Tedford *et al.* used a GST-fusion for the production of an insecticidal spider toxin containing three disulfide bonds [63]. Our present work shows that many other DRPs can be produced and purified to homogeneity at the milligram scale using our protocols, spanning a diversity of folds (α/β, ICK, Kazal type, Kunitz). We observed that some proteins tested were more prone to adopt their oxidized native conformation than others. As an illustration, we can see that mCD4M61, Psalmotoxin-1 and LDTI were detected in MALDI-TOF analyses in virtually all cases, independently of the fusion partner used. On the contrary, EVIA and ShK toxins were only detected in a few conditions tested. In addition, yields from large-scale expression of DRPs were quite case-dependent, ranging from a few micrograms for the hard-to-express muscarinic toxin MT7 containing four disulfide bonds to more than 12 mg for LDTI. In some cases, the amounts of DRPs obtained after all production and purification steps are lower than what could be expected from the amounts of fusion protein produced. It has to be noted that no individual optimization was performed to increase yield for individual DRPs. Thus, one could reasonably expect to increase yields by improving the process for a specific protein (buffers, purification, choice of the protease) or by performing further *in vitro* refolding steps.

While this optimization would be an interesting option to increase yields, one should also consider other tools to encourage increased efficiency of correct peptide folding. Indeed, the ratios between purified DRPs versus purified fusion proteins suggest that the folding of some proteins is particularly incomplete (e.g. MT7, Thrombin Inhibitor Infestin or Trypsin Inhibitor II). Among the further improvements envisioned, it may be of interest to express concomitantly and in stoichiometric excess a second redox-active protein (oxidase or isomerase) to boost the positive effect of DsbC. Besides that, the lysis buffer components could possibly have an influence on the DRP folding. Given the hypothesis of an *ex vivo* oxidation it would be of interest to investigate the effect of introducing oxidized/reduced glutathione which are classically used for *in vitro* DRP refolding strategies. Additionally, use of tools promoting the *in vivo* folding and oxidation of the DRP would be an interesting option to achieve higher productivity. Co-expression of sulfhydryl oxidases [61,62,65] or addition of redox molecules in the medium [66] could be promising options to promote the formation of disulfides bonds *in vivo*. These tools could be additionally introduced in our screen without significant modification of the process.

## Conclusion

Our high throughput screening approach allows the systematic investigation of the solubilizing and folding influence of various partners in the strains BL21 (DE3) pLysS, Origami B (DE3) pLysS and SHuffle® T7 Express lysY for the production of soluble DRPs. In spite of its reducing cytoplasm, BL21 (DE3) pLysS is a very efficient strain for the production of DRPs in fusion with solubilizing partners. In our set-up, *trxB*^*-*^*/gor*^*-*^ strains Origami B (DE3) pLysS and SHuffle® T7 Express lysY yielded only very low amounts of fusion proteins. Many DRPs are found oxidized after production in BL21 (DE3) pLysS, most probably because of post-lytic oxidation reactions. In many ways, use of DsbC as a fusion partner in the strain BL21 (DE3) pLysS was the most potent combination tested. Our protocols allow the production of a large diversity of DRPs using DsbC as a fusion partner, leading to pure active DRPs at milligram scale in many cases. Thus, this work should facilitate the study of DRPs with therapeutic or biotechnological interest whose production was previously a limiting step.

## Materials and methods

### Design and construction of the expression plasmid library

Synthetic genes optimized for recombinant expression of miniproteins in *E. coli* were ordered from Geneart AG. These genes contain the sequence coding for a TEV protease cleavage site (ENLYFQ/G) followed by the sequence corresponding to the DRP (see Additional file 1: Table S1), with Gateway recombination sites on each extremity of the gene. These synthetic constructs were cloned by Gateway™ BP cloning technology using pDONR221 as a donor vector. Twelve Gateway destination vectors were used in this study. Each contains one of the eleven fusion partners and a 6HIS tag for protein purification located either on the N- or C-terminal side of the fusion partner and the twelfth vector containing 6HIS alone (see Additional file 2: Table S2). The 28 entry clones were in turn recombined using the Gateway™ LR cloning technology using one of the 12 Gateway destination vectors. Thus, a total of 336 different expression plasmids were created. All cloning steps (Gateway LR cloning of the 28 pENTR clones in 12 destination vectors, DNA purifications, bacterial transformations and cultures) were accomplished within a week using high throughput compliant protocols detailed elsewhere [9].

### High throughput protein expression screening

In this study, all cultures were grown in auto-induction medium ZYP-5052 supplemented with antibiotics both for small-scale expression screens (in BL21 (DE3) pLysS, Origami (DE3) pLysS and SHuffle® T7 Express lysY) or scale-up expression (in BL21 (DE3) pLysS). ZYP-5052 medium is a buffered complex medium containing glucose, lactose and glycerol formulated to induce protein expression after glucose depletion [50]. Expressions were performed using a standardized two-step process. In the first part of fermentation, cells were grown at 37°C to quickly reach the glucose depletion phase just before the induction. After that step (4 hours) the temperature was lowered to 17°C for 18h to favor protein folding and soluble protein expression.

All steps were carried out in 24 or 96 deep-well plates (DW24 and DW96, respectively). Expression strains were obtained after a heat-shock transformation of competent cells with the expression plasmids. Transformed cells were used to inoculate pre-cultures in DW96 plates containing 1 mL of LB media in each well. The following morning, 100 μL of the pre-culture broth was used to inoculate 4 mL of ZYP-5052 medium. Cultivation was carried out using DW24 plates to increase the biomass compared to DW96 cultures. After an overnight incubation at 17°C, cells were pelleted by centrifugation, resuspended in lysis buffer and transferred into DW96 and frozen at −80°C. After thawing the cells the lysate was purified using an automated nickel affinity procedure as described in Figure 2. The whole procedure for the BL21 (DE3) pLysS and Origami B (DE3) pLysS (672 cultures followed by purification and analysis), was performed within a week and reproduced a second time to confirm the results (these protocols have been detailed elsewhere [9,49]) while the SHuffle® T7 Express lysY experiment was done on a separate week.

### Identification of soluble expression conditions

In this study, the analysis of the purified protein yields (as well as the efficiency of TEV cleavage) was performed on a Labchip GXII (Caliper, USA) microfluidic high throughput system, which was more adapted to the throughput of this work than traditional SDS-PAGE analysis. This analysis (done following the manufacturer’s instructions) provides an estimation of the molecular weight, concentration and purity of the proteins with a detection limit of approximately 0.1 mg/L of culture. Proteins below 5 kDa could not be assessed by this method due to system limits. For the DRPs below this molecular weight, the cleavage efficiency was initially assessed by the disappearance of the fusion-DRP species. The molecular weight of the free DRP was only confirmed by mass spectrometry at scale up.

### Large-scale cultures, purification and cleavage of DsbC-DRPs

For larger scale production of recombinant proteins, a Fernbach flask containing 1 L of ZYP-5052 auto-induction medium and the appropriate antibiotics was inoculated with an overnight culture of BL21 (DE3) pLysS to reach 0.05 O.D. at 600 nm. Cultures were performed using the two-step protocol (4h at 37°C then 18h at 17°C). At the end of culture, cells were then harvested by centrifugation (4500 × *g*, 30 min, 4°C) and the pellet was resuspended in 50 mL of lysis buffer (100 mM Tris–HCl, pH 8, 150 mM NaCl, 5% glycerol). Lysis of cells was performed using a cell disrupter (Constant System Ltd). The lysate was cleared by centrifugation (18 000 rpm, 30 min, 4°C) and the supernatant loaded onto a 5 mL HisTrap FF column (GE-Healthcare Bio-Sciences). The 6HIS-tagged fusion proteins were then eluted with a linear gradient (0 to 100% B in 30 min at a flow rate of 2 mL/min) of buffer B (100 mM Tris–HCl, pH 8, 150 mM NaCl, 5% glycerol, 500 mM imidazole) in buffer A (100 mM Tris–HCl, pH 8, 150 mM NaCl, 5% glycerol, 40 mM imidazole). The fractions containing the 6HIS-tagged fusion protein were pooled and dialyzed for 3h against lysis buffer using a Spectra/Por® Dialysis Membrane (MWCO: 3500). The protein of interest was then cleaved with 10% (w/w) TEV protease overnight at 4°C and purified by RP-HPLC. RP-HPLC purification was performed using a semi-preparative C4 column (Vydac 214TP1010, 10 μm, 300 Å, 10 × 250 mm) using a linear gradient 0-60% in 30 min of solvent B (100% acetonitrile, 0.09% TFA) in solvent A (100% H_2_O, 0.1% TFA) with a flow rate of 4 mL/min. After HPLC purification, DRPs were lyophilized and solubilized in the appropriate buffer for further studies.

### Characterization by mass spectrometry

Detection of oxidized DRPs used MALDI-TOF and LTQ-Orbitrap. The purified fusion protein samples were digested by TEV protease at 4°C. Samples were loaded onto C18 reverse phase ZipTips, desalted and eluted by 70% acetonitrile/ H_2_O /0.1% TFA before spotting on a MALDI plate with 4-CHCA matrix at 10 mg/mL. MALDI-TOF analyses were performed on a MALDI-TOF/TOF™ 4800 Analyzer from AB-SCIEX (Foster City, CA). The isotopic pattern measured was compared with the theoretical one determined from the amino acid sequences using DataExplorer software (Version 4.9, Applied Biosystems).

High resolution/high mass accuracy measurements were performed on an LTQ-Orbitrap instrument (Thermo, San Jose, CA) by UHPLC-MS essentially as described previously [67]. Briefly, DRP samples were loaded and separated on a C18 Hypersil GOLD column (2.1 mm x 150 mm, 175 Å, 1.9 μm, ThermoScientific) at a flow rate of 300 μL/min with a linear gradient of 0 to 80% B in 10 min (with solvent A: H_2_O containing 0.1% formic acid and solvent B: acetonitrile containing 0.1% formic acid). MS acquisition was performed in the positive ion mode from *m/z* 500 to 2000 using a resolution set at 30000 (at *m/z* 400). The resulting mass spectra were deconvoluted using ProMass software (ThermoScientific).

### Functional characterization of purified DRPs

Enzyme inhibition by the inhibitors was tested in competition experiments. For plasmin tests, we used 6 μM fluorogenic substrate N-succinimyl-Ala-Phe-Lys-AMC (Sigma Aldrich) and plasmin (Sigma Aldrich) at 0.1 nM concentration in 100 μL of PBS buffer. For Trypsin tests, we used 1.8 μM fluorogenic substrate Mca-R-P-K-P-V-E-NVal-W-R-P-K(Dnp)-NH2 (R&D Systems) and Trypsin (Sigma Aldrich) at 0.4 nM concentration in 200 μL of 50 mM Tris–HCl buffer pH 6.8, 100 mM NaCl, 10 mM CaCl_2_.

The substrate and enzyme concentrations for the experiments were chosen so as to remain well below 10% of substrate utilization and to observe the initial rates. For each inhibitor, the percentage of inhibition was determined in duplicate experiments at four inhibitor concentrations, chosen to observe a 20–80% range of inhibition. After 1h incubation with shaking at 25°C, inhibition assays were performed by recording the fluorescence increase induced by the cleavage of fluorogenic substrate, using 96-well nonbinding surface plates (3650 Corning-Costar plates). Fluorescence signals were monitored using a Fluoroscan Ascent photon counter spectrophotometer (ThermolabSystems) equipped with a temperature control device and a plate shaker. K_i_ values were determined using the method proposed by Horovitz and Levitski [68].

The property of miniCD4 (mCD4M61) to inhibit the binding of HIV-envelope was evaluated by *in vitro* competitive ELISA as previously described [3]. The effect of MT7 on the equilibrium bonding of [3H]NMS on M1 muscarinic receptor was determined in inhibition experiments as previously described [51].

## Abbreviations

BPTI: Bovine pancreatic trypsin inhibitor; LDTI: Leech-Derived Tryptase Inhibitor; DRP: Disulfide-rich proteins; ICK: Inhibitor Cystine Knot; 3FT: Three-finger toxin; TDPI: Tick-derived Protease Inhibitor; RBI: Ragi bifunctional inhibitor; MALDI-TOF: Matrix-assisted laser desorption/ionization-Time of flight.

## Competing interests

The authors declare that they have no competing interests.

## Authors’ contributions

HN, RV, DS, MG, PC and VD have set up and designed the study; HN and MM cloned the destination plasmids; RV designed and supervised the high throughput experiments; HN, RV, NS have cloned the expression plasmids and performed high throughput screening recombinant expression experiments; HN, CFG, OHPR, FB and BD have performed scale-up expression, purification and characterization of selected DRPs. HN, FF and RT have performed mass spectroscopy experiments; HN, RV and VD have analyzed the data and written the manuscript; all authors discussed the results and commented on the manuscript and all authors read and approved the final manuscript.

## Supplementary Material

Additional file 1: Table S1 Disulfide-rich proteins and their characteristics. Sequences beginning with SSCS have a modified N-terminus sequence, however the cysteine position and pattern has been conserved. Click here for file

Additional file 2: Table S2 Destination plasmids used in this study. Click here for file

Additional file 3: Table S3 Molecular masses of the 25 DsbC-DRP fusions as determined by LTQ-Orbitrap mass spectrometer. Click here for file

## References

[B1] King GF Venoms as a platform for human drugs: translating toxins into therapeutics Expert Opin Biol Ther 2011 11 1469 1484 10.1517/14712598.2011.621940 21939428

[B2] Miljanich GP Ziconotide: Neuronal calcium channel blocker for treating severe chronic pain Curr Med Chem 2004 11 3029 3040 10.2174/0929867043363884 15578997

[B3] Martin L Stricher F Misse D Sironi F Pugniere M Barthe P Prado-Gotor R Freulon I Magne X Roumestand C Rational design of a CD4 mimic that inhibits HIV-1 entry and exposes cryptic neutralization epitopes Nat Biotechnol 2003 21 71 76 12483221 10.1038/nbt768

[B4] Smith GP Patel SU Windass JD Thornton JM Winter G Griffiths AD Small binding proteins selected from a combinatorial repertoire of knottins displayed on phage J Mol Biol 1998 277 317 332 10.1006/jmbi.1997.1621 9514763

[B5] Hancock REW Sahl HG Antimicrobial and host-defense peptides as new anti-infective therapeutic strategies Nat Biotechnol 2006 24 1551 1557 10.1038/nbt1267 17160061

[B6] Kimura RH Cheng Z Gambhir SS Cochran JR Engineered knottin peptides: a New class of agents for imaging integrin expression in living subjects Cancer Res 2009 69 2435 2442 10.1158/0008-5472.CAN-08-2495 19276378 PMC2833353

[B7] Demain AL Vaishnav P Production of recombinant proteins by microbes and higher organisms Biotechnol Adv 2009 27 297 306 19500547 10.1016/j.biotechadv.2009.01.008

[B8] Braud S Moutiez M Belin P Abello N Drevet P Zinn-Justin S Courcon M Masson C Dassa J Charbonnier JB Dual expression system suitable for high-throughput fluorescence-based screening and production of soluble proteins J Proteome Res 2005 4 2137 2147 10.1021/pr050230i 16335960

[B9] Vincentelli R Cimino A Geerlof A Kubo A Satou Y Cambillau C High-throughput protein expression screening and purification in Escherichia coli Methods 2011 55 65 72 10.1016/j.ymeth.2011.08.010 21925268

[B10] Groisillier A Herve C Jeudy A Rebuffet E Pluchon PF Chevolot Y Flament D Geslin C Morgado IM Power D MARINE-EXPRESS: taking advantage of high throughput cloning and expression strategies for the post-genomic analysis of marine organisms Microb Cell Fact 2010 9 45 10.1186/1475-2859-9-45 20546566 PMC2897777

[B11] Xiao R Anderson S Aramini J Belote R Buchwald WA Ciccosanti C Conover K Everett JK Hamilton K Huang YJ The high-throughput protein sample production platform of the Northeast Structural Genomics Consortium J Struct Biol 2010 172 21 33 10.1016/j.jsb.2010.07.011 20688167 PMC4110633

[B12] Douzi BGA Gilles N Darbon H Marchot P Vincentelli R Barbier JBE Marchot P Mattei C Servent D A new system for expressing recombinant animal toxins in E. coli Advances and new technologies in toxinology 2010 France: Collection Rencontres en Toxinologie, Publications de la SFET, Châtenay-Malabry 149 152

[B13] Bogomolovas J Simon B Sattler M Stier G Screening of fusion partners for high yield expression and purification of bioactive viscotoxins Protein Expr Purif 2009 64 16 23 10.1016/j.pep.2008.10.003 18983922

[B14] Chen YQ Zhang SQ Li BC Qiu W Jiao B Zhang J Diao ZY Expression of a cytotoxic cationic antibacterial peptide in Escherichia coli using two fusion partners Protein Expr Purif 2008 57 303 311 10.1016/j.pep.2007.09.012 17977015

[B15] Torres FS Silva CN Lanza LF Santos AV Pimenta AM De Lima ME Diniz MR Functional expression of a recombinant toxin - rPnTx2-6 - active in erectile function in rat Toxicon 2010 56 1172 1180 10.1016/j.toxicon.2010.04.010 20417652

[B16] Lyukmanova EN Shulepko MA Shenkarev ZO Dolgikh DA Kirpichnikov MP In vitro production of three-finger neurotoxins from snake venoms, a disulfide rich proteins. Problems and their solutions (Review) Russ J Bioorg Chem 2010 36 137 145 10.1134/S1068162010020019 20531472

[B17] de Marco A Strategies for successful recombinant expression of disulfide bond-dependent proteins in Escherichia coli Microb Cell Fact 2009 8 26 10.1186/1475-2859-8-26 19442264 PMC2689190

[B18] Weissman JS Kim PS Reexamination of the folding of BPTI: predominance of native intermediates Science 1991 253 1386 1393 10.1126/science.1716783 1716783

[B19] Creighton TE The disulfide folding pathway of BPTI Science 1992 256 111 114 10.1126/science.1373519 1373519

[B20] Chang JY The disulfide folding pathway of tick anticoagulant peptide (TAP), a Kunitz-type inhibitor structurally homologous to BPTI Biochemistry 1996 35 11702 11709 10.1021/bi9606915 8794751

[B21] Bronsoms S Pantoja-Uceda D Gabrijelcic-Geiger D Sanglas L Aviles FX Santoro J Sommerhoff CP Arolas JL Oxidative folding and structural analyses of a kunitz-related inhibitor and its disulfide intermediates: functional implications J Mol Biol 2011 414 427 441 10.1016/j.jmb.2011.10.018 22033478

[B22] de Marco A Vigh L Diamant S Goloubinoff P Native folding of aggregation-prone recombinant proteins in Escherichia coli by osmolytes, plasmid- or benzyl alcohol-overexpressed molecular chaperones Cell Stress Chaperones 2005 10 329 339 10.1379/CSC-139R.1 16333986 PMC1283876

[B23] Baneyx F Mujacic M Recombinant protein folding and misfolding in Escherichia coli Nat Biotechnol 2004 22 1399 1408 10.1038/nbt1029 15529165

[B24] Saez NJ Mobli M Bieri M Chassagnon IR Malde AK Gamsjaeger R Mark AE Gooley PR Rash LD King GF A dynamic pharmacophore drives the interaction between Psalmotoxin-1 and the putative drug target acid-sensing ion channel 1a Mol Pharmacol 2011 80 796 808 10.1124/mol.111.072207 21825095

[B25] Anangi R Rash LD Mobli M King GF Functional expression in escherichia coli of the disulfide-rich sea anemone peptide APETx2, a potent blocker of acid-sensing Ion channel 3 Mar Drugs 2012 10 1605 1618 10.3390/md10071605 22851929 PMC3407934

[B26] Heras B Kurz M Shouldice SR Martin JL The name's bond......disulfide bond Curr Opin Struct Biol 2007 17 691 698 10.1016/j.sbi.2007.08.009 17933514

[B27] Ito K Inaba K The disulfide bond formation (Dsb) system Curr Opin Struct Biol 2008 18 450 458 10.1016/j.sbi.2008.02.002 18406599

[B28] Fernandez C Salinas G Pellizza L Margenat M Flo M Tuned Escherichia coli as a host for the expression of disulfide-rich proteins Biotechnol J 2011 6 686 699 10.1002/biot.201000335 21567960

[B29] Yoon SH Kim SK Kim JF Secretory production of recombinant proteins in escherichia coli Recent Pat Biotechnol 2010 4 23 29 10.2174/187220810790069550 20201800

[B30] Bessette PH Aslund F Beckwith J Georgiou G Efficient folding of proteins with multiple disulfide bonds in the Escherichia coli cytoplasm Proc Natl Acad Sci USA 1999 96 13703 13708 10.1073/pnas.96.24.13703 10570136 PMC24128

[B31] Derman AI Prinz WA Belin D Beckwith J Mutations that allow disulfide bond formation in the cytoplasm of Escherichia-coli Science 1993 262 1744 1747 10.1126/science.8259521 8259521

[B32] Xu Y Yasin A Tang R Scharer JM Moo-Young M Chou CP Heterologous expression of lipase in Escherichia coli is limited by folding and disulfide bond formation Appl Microbiol Biotechnol 2008 81 79 87 10.1007/s00253-008-1644-6 18758768

[B33] Saejung W Puttikhunt C Prommool T Sojikul P Tanaka R Fujiyama K Malasit P Seki T Enhancement of recombinant soluble dengue virus 2 envelope domain III protein production in Escherichia coli trxB and gor double mutant J Biosci Bioeng 2006 102 333 339 10.1263/jbb.102.333 17116581

[B34] Faulkner MJ Veeravalli K Gon S Georgiou G Beckwith J Functional plasticity of a peroxidase allows evolution of diverse disulfide-reducing pathways Proc Natl Acad Sci USA 2008 105 6735 6740 10.1073/pnas.0801986105 18456836 PMC2373332

[B35] Skretas G Carroll S DeFrees S Schwartz MF Johnson KF Georgiou G Expression of active human sialyltransferase ST6GalNAcI in Escherichia coli Microb Cell Fact 2009 8 50 10.1186/1475-2859-8-50 19788761 PMC2762462

[B36] Lobstein J Emrich CA Jeans C Faulkner M Riggs P Berkmen M SHuffle, a novel Escherichia coli protein expression strain capable of correctly folding disulfide bonded proteins in its cytoplasm Microb Cell Fact 2012 11 56 10.1186/1475-2859-11-56 22569138 PMC3526497

[B37] Jonathan Beckwith FA Paul H Bessette George G Daniel R Lim JEa United states: President and Fellows of Harvard College, Cambridge, MA (US; Board of Regents Compositions and methods for production of disulfide bond containing proteins in host cells 2008 Austin, TX (US): University of Texas System

[B38] Sorensen HP Mortensen KK Soluble expression of recombinant proteins in the cytoplasm of Escherichia coli Microb Cell Fact 2005 4 1 10.1186/1475-2859-4-1 15629064 PMC544838

[B39] Esposito D Chatterjee DK Enhancement of soluble protein expression through the use of fusion tags Curr Opin Biotechnol 2006 17 353 358 10.1016/j.copbio.2006.06.003 16781139

[B40] Walls D Loughran ST Tagging recombinant proteins to enhance solubility and aid purification Methods Mol Biol 2011 681 151 175 10.1007/978-1-60761-913-0_9 20978965

[B41] Smith DB Johnson KS Single-step purification of polypeptides expressed in escherichia-coli as fusions with glutathione S-transferase Gene 1988 67 31 40 10.1016/0378-1119(88)90005-4 3047011

[B42] Maina CV Riggs PD Grandea AG 3rd Slatko BE Moran LS Tagliamonte JA McReynolds LA Guan CD An Escherichia coli vector to express and purify foreign proteins by fusion to and separation from maltose-binding protein Gene 1988 74 365 373 10.1016/0378-1119(88)90170-9 3073105

[B43] Drevet P Lemaire C Gasparini S ZinnJustin S Lajeunesse E Ducancel F Pinkasfeld S Courcon M Tremeau O Boulain JC Menez A High-level production and isotope labeling of snake neurotoxins, disulfide-rich proteins Protein Expr Purif 1997 10 293 300 10.1006/prep.1997.0740 9268675

[B44] Huth JR Bewley CA Jackson BM Hinnebusch AG Clore GM Gronenborn AM Design of an expression system for detecting folded protein domains and mapping macromolecular interactions by NMR Protein Sci 1997 6 2359 2364 9385638 10.1002/pro.5560061109 PMC2143577

[B45] Dyson MR Shadbolt SP Vincent KJ Perera RL McCafferty J Production of soluble mammalian proteins in Escherichia coli: identification of protein features that correlate with successful expression BMC Biotechnol 2004 4 32 10.1186/1472-6750-4-32 15598350 PMC544853

[B46] Jurado P Ritz D Beckwith J de Lorenzo V Fernandez LA Production of functional single-chain Fv antibodies in the cytoplasm of Escherichia coli J Mol Biol 2002 320 1 10 10.1016/S0022-2836(02)00405-9 12079330

[B47] Gleiter S Bardwell JC Disulfide bond isomerization in prokaryotes Biochim Biophys Acta 2008 1783 530 534 10.1016/j.bbamcr.2008.02.009 18342631 PMC2391271

[B48] Marsischky G LaBaer J Many paths to many clones: a comparative look at high-throughput cloning methods Genome Res 2004 14 2020 2028 10.1101/gr.2528804 15489321

[B49] Saez NJ Vincentelli R High-throughput expression screening and purification of recombinant proteins in E. coli Methods Mol Biol 2013 in press 10.1007/978-1-62703-691-7_3 24203323

[B50] Studier FW Protein production by auto-induction in high density shaking cultures Protein Expr Purif 2005 41 207 234 10.1016/j.pep.2005.01.016 15915565

[B51] Fruchart-Gaillard C Mourier G Marquer C Ménez A Servent D Identification of various allosteric interaction sites on M1 muscarinic receptor using 125I-Met35-oxidized muscarinic toxin 7 Mol Pharmacol 2006 69 1641 1651 10.1124/mol.105.020883 16439611

[B52] Campos ITN Amino R Sampaio CAM Auerswald EA Friedrich T Lemaire HG Schenkman S Tanaka AS Infestin, a thrombin inhibitor presents in Triatoma infestans midgut, a Chagas' disease vector: gene cloning, expression and characterization of the inhibitor Insect Biochem Mol Biol 2002 32 991 997 10.1016/S0965-1748(02)00035-8 12213235

[B53] Heitz A Avrutina O Le-Nguyen D Diederichsen U Hernandez JF Gracy J Kolmar H Chiche L Knottin cyclization: impact on structure and dynamics BMC Struct Biol 2008 8 54 10.1186/1472-6807-8-54 19077275 PMC2659701

[B54] Stubbs MT Morenweiser R Sturzebecher J Bauer M Bode W Huber R Piechottka GP Matschiner G Sommerhoff CP Fritz H Auerswald EA The three-dimensional structure of recombinant leech-derived tryptase inhibitor in complex with trypsin - Implications for the structure of human mast cell tryptase and its inhibition J Biol Chem 1997 272 19931 19937 10.1074/jbc.272.32.19931 9242660

[B55] Grzesiak A Helland R Smalas AO Krowarsch D Dadlez M Otlewski J Substitutions at the P-1 ' position in BPTI strongly affect the association energy with serine proteinases J Mol Biol 2000 301 205 217 10.1006/jmbi.2000.3935 10926503

[B56] Berkmen M Production of disulfide-bonded proteins in Escherichia coli Protein Expr Purif 2012 82 240 251 10.1016/j.pep.2011.10.009 22085722

[B57] Lefebvre J Boileau G Manjunath P Recombinant expression and affinity purification of a novel epididymal human sperm-binding protein, BSPH1 Mol Hum Reprod 2009 15 105 114 19091820 10.1093/molehr/gan077

[B58] Tait AR Straus SK Overexpression and purification of U24 from human herpesvirus type-6 in E. coli: unconventional use of oxidizing environments with a maltose binding protein-hexahistine dual tag to enhance membrane protein yield Microb Cell Fact 2011 10 51 10.1186/1475-2859-10-51 21714924 PMC3155487

[B59] Saaranen MJ Ruddock LW Disulfide bond formation in the cytoplasm Antioxid Redox Signal 2012 in press 10.1089/ars.2012.4868 22870953

[B60] Xiong S Wang YF Ren XR Li B Zhang MY Luo Y Zhang L Xie QL Su KY Solubility of disulfide-bonded proteins in the cytoplasm of Escherichia coli and its "oxidizing" mutant World J Gastroenterol 2005 11 1077 1082 15742420 10.3748/wjg.v11.i7.1077 PMC4250777

[B61] Hatahet F Nguyen VD Salo KE Ruddock LW Disruption of reducing pathways is not essential for efficient disulfide bond formation in the cytoplasm of E. coli Microb Cell Fact 2010 9 67 20836848 10.1186/1475-2859-9-67 PMC2946281

[B62] Nguyen VD Hatahet F Salo KE Enlund E Zhang C Ruddock LW Pre-expression of a sulfhydryl oxidase significantly increases the yields of eukaryotic disulfide bond containing proteins expressed in the cytoplasm of E.coli Microb Cell Fact 2011 10 1 10.1186/1475-2859-10-1 21211066 PMC3022669

[B63] Tedford HW Fletcher JI King GF Functional significance of the beta-hairpin in the insecticidal neurotoxin omega-atracotoxin-Hv1a J Biol Chem 2001 276 26568 26576 10.1074/jbc.M102199200 11313356

[B64] Mac TT Beyermann M Pires JR Schmieder P Oschkinat H High yield expression and purification of isotopically labelled human endothelin-1 for use in NMR studies Protein Expr Purif 2006 48 253 260 10.1016/j.pep.2006.01.022 16584890

[B65] Abskharon RN Ramboarina S El Hassan H Gad W Apostol MI Giachin G Legname G Steyaert J Messens J Soror SH Wohlkonig A A novel expression system for production of soluble prion proteins in E. coli Microb Cell Fact 2012 11 6 10.1186/1475-2859-11-6 22233534 PMC3283519

[B66] Beld J Woycechowsky KJ Hilvert D Small-molecule diselenides catalyze oxidative protein folding in vivo ACS Chem Biol 2010 5 177 182 10.1021/cb9002688 20052969

[B67] Contrepois K Ezan E Mann C Fenaille F Ultra-high performance liquid chromatography-mass spectrometry for the fast profiling of histone post-translational modifications J Proteome Res 2010 9 5501 5509 10.1021/pr100497a 20707390

[B68] Horovitz A Levitzki A An accurate method for determination of receptor-ligand and enzyme-inhibitor dissociation constants from displacement curves Proc Natl Acad Sci USA 1987 84 6654 6658 10.1073/pnas.84.19.6654 3477796 PMC299141

